# Gait speed and cognitive decline over 2 years in the Ibadan study of aging

**DOI:** 10.1016/j.gaitpost.2015.01.011

**Published:** 2015-02

**Authors:** Akin Ojagbemi, Catherine D’Este, Emese Verdes, Somnath Chatterji, Oye Gureje

**Affiliations:** a WHO Collaborating Centre for Research and Training in Mental Health, Neuroscience and Substance Abuse, Department of Psychiatry, College of Medicine, University of Ibadan, P.M.B. 5017 (GPO), Ibadan, Nigeria; b National Centre for Epidemiology and Population Health, Research School of Population Health, ANU College of Medicine, Biology and Environment, The Australian National University, Building 62 Mills Road, Canberra ACT 0200, Australia; c Department of Health Statistics and Informatics at the World Health Organization (WHO), 20 Avenue Appia, Geneva 1207 CH-1211, Switzerland

**Keywords:** Gait speed, Cognition, Dementia, Developing countries

## Abstract

**Background:**

The evidence suggesting that gait speed may represent a sensitive marker for cognitive decline in the elderly requires support from diverse racial groups.

**Objective:**

We investigated the relationship between gait speed and cognitive decline over 2 years in a community dwelling sample of elderly Africans.

**Methods:**

Data are from the Ibadan study of aging (ISA) conducted among a household multi-stage probability sample of 2149 Yoruba Nigerians aged 65 years or older. Gait speed was measured as the time taken to complete a 3 or 4 m distance at normal walking speed. We assessed cognitive functions with a modified version of the 10-word learning list and delay recall test, and examined the relationship between baseline gait speed, as well as gait speed changes, and follow-up cognition using multiple linear regression and longitudinal analyses using random effects.

**Results:**

Approximately 71% of 1461 participants who were dementia free and who had their gait speed measured at baseline (2007) were successfully followed up in two waves (2008 and 2009). Along with increasing age, poor health and economic status, a slower baseline gait speed was independently associated with poorer follow-up cognition in both linear regression (1.2 words, 95% CI = 0.48–2.0) and longitudinal analyses (0.8 words, 95% CI = 0.44–1.2). Also, a greater change in gait speed between 2007 and 2009 was associated with the worst follow-up cognition (0.3 words, 95% CI = 0.09–0.51).

**Conclusion:**

The finding that a substantial change in gait speed was associated with reduced cognitive performance is of potential importance to efforts aimed at early identification of cognitive disorders in this population.

## Highlights

• We studied the elderly in an area populated by a quarter of Nigerians.
• Gait speed was associated with the average follow-up cognition.
• Gait speed was associated with longitudinal changes in cognition.
• Follow-up cognition score was associated with gait speed change over 2 years.

## 1 Background

Studies conducted in Western Europe and North America have reported that gait speed is an important indicator of global functioning and health of the elderly [1,2]. Gait mechanics involve the interplay of many cognitive and motor functions known to decline with aging [1,3]. In line with this relationship, clinico-pathological correlations [4] and prospective longitudinal studies [5] suggest that gait speed dysfunction is associated with early markers of cognitive disorders in older adults.

In a large prospective longitudinal study of community dwelling elderly Nigerians we aimed to: (1) determine whether baseline gait speed was associated with cognitive function at 2-year follow-up; (2) determine whether there was an association between rate of change in gait speed and cognitive function at follow-up; and (3) assess the relationship between change in gait speed and change in cognition over time.

## 2 Methods

### 2.1 Sample

The Ibadan study of aging [6] is a longitudinal cohort study of persons aged 65 years and over selected using stratified multistage cluster sampling from eight neighboring states of the Yoruba-speaking region of Nigeria.

Wave one of the data collection was conducted between November 3, 2003 and August 27, 2004; with follow up in 2007 (including 552 additional households), 2008 and 2009. The present report is based on assessments conducted in 2007 (as baseline) and 2009 (as follow-up).

### 2.2 Measures

The cognition outcome was the total number of words correctly recalled for the third repetition of the 10 word recall test and the 10-word delay recall test, to reflect both learning and retention capabilities [7].

Gait speed was measured, using a digital stopwatch, as the time to the nearest one-hundredth of a second for participants to walk a distance of 3 or 4 m, with the distance dependant on their general level of physical function and ability. The test was undertaken twice with the shortest time taken as the final measure. The time was classified as [2]: 0 if the participant was unable to do the walk; 1 if >8.70 s (4 m walk) or >6.52 s (3 m walk); 2 if 6.21–8.70 s (4 m walk) or 4.66–6.52 s (3 m walk); 3 if 4.82–6.20 s (4 m walk) or 3.62–4.65 s (3 m walk) or 4 if <4.82 s (4 m walk) or <3.62 s (3 m walk), with the lower two categories combined.

### 2.3 Statistical methods

Participants with a dementia diagnosis at baseline, determined by psychiatrist review, were excluded. We reversed cognition score and gait speed categories so that higher values indicated worse scores. We then undertook linear regression analyses with word recall measure in 2009 as the outcome and gait speed in 2007 as an explanatory variable, and adjusted for baseline cognition score. Further, we separately included change in gait speed category from 2007 to 2008 and from 2007 to 2009. These analyses used the survey commands in Stata [Stata Corp] to account for the sampling scheme. Finally, we undertook a random effects longitudinal analysis of observations for 2007, 2008 and 2009 using the xtmixed commands in Stata with robust standard errors. All regression models included socio-demographic, self-reported health and major depressive disorder within the previous 12 months [8,9]. Lifestyle risk factors and chronic conditions were ascertained using standard symptom-based questions [10], while hypertension and BMI category based on measured values [11] are presented for longitudinal analysis only as these variables had minimal impact in cross-sectional analyses.

We undertook sensitivity analyses using multiple imputation to adjust for missing data.

### 2.4 Ethical approval

Ethical approval was obtained from the University of Ibadan and University College Hospital, Ibadan Joint Ethical Review Board.

## 3 Results

**Fig. 1:**
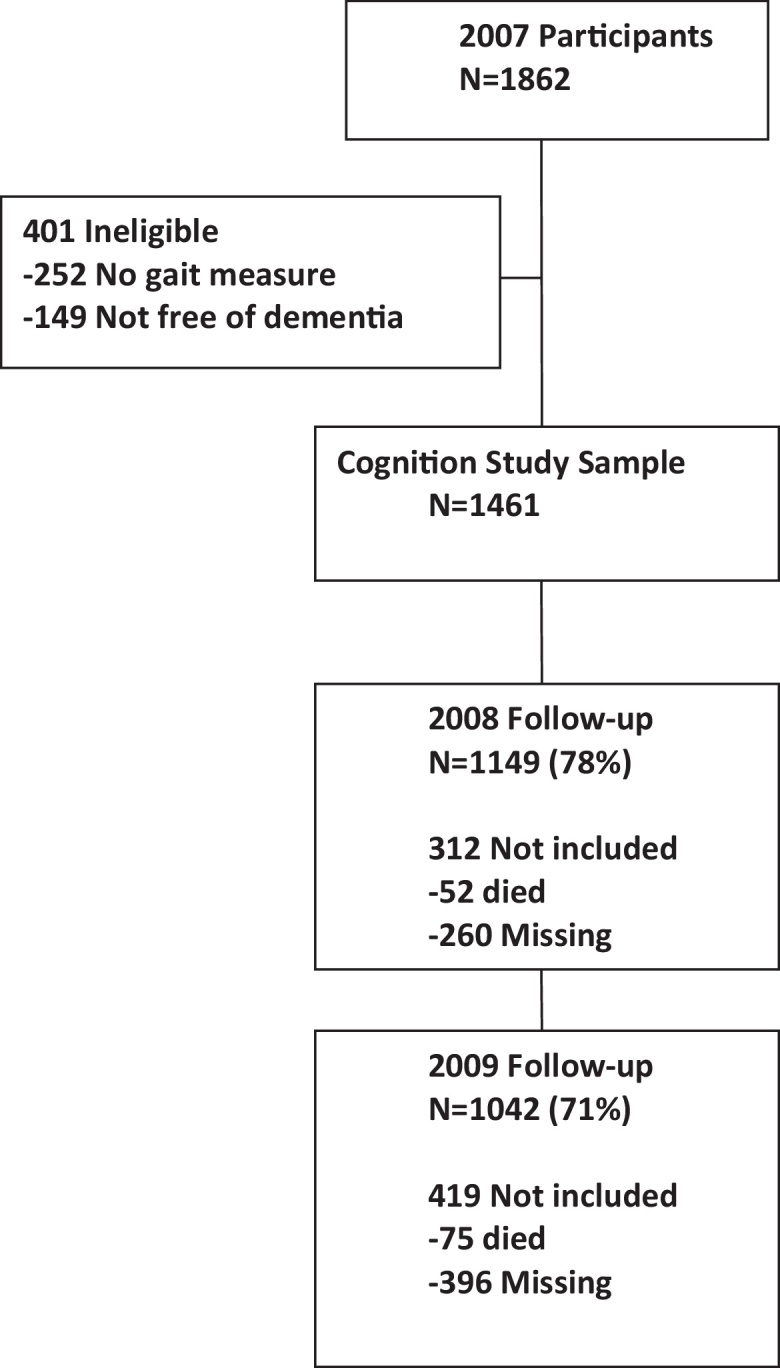
Flow chart for the study.

Of the 1461 individuals in the 2007 cohort, 1042 were followed-up in 2009 (Fig. 1); follow-up was associated with younger age, being married, and higher baseline word recall score and gait speed.

Word recall declined with poorer gait speed (Table 1), with a difference in adjusted mean follow-up word recall of almost 1.25 words between the best and worst gait speed categories. A decline of one category in gait speed between 2007 and 2009, but not 2007–2008 was associated with a reduction in word recall of approximately 0.3 of a word.

In longitudinal analyses word recall score declined over time and worse gait score remains associated with poorer recall (Table 2). Analyses following multiple imputation demonstrated similar results to complete case analyses.

## 4 Discussion

We report a relationship between baseline gait speed and cognition at follow-up for up to 2 years for both average follow-up cognition scores and longitudinal changes in cognition. Our analyses also revealed an association between follow-up cognition score and change in gait speed between 2007 and 2009 but not for the shorter period of 2007–2008.

Our findings are consistent with several reports, mostly from Western Europe and America, of a relationship between gait speed and cognition [5,12]. However we note that factors such as a lower level of Western education, higher rates of depression [6], and variable degrees of cognitive stimulation [13] in the elderly living in the country setting of this study may influence performance in the available cognitive tests. Also, poor health and a lower socio-economic status which are common in the elderly, especially those living in poorer countries, and which were associated with impaired follow-up word recall in this study, have also been shown to be associated with the incidence of cognitive disorders in the study setting [13].

The finding that change in gait speed over 2 years was a significant predictor of follow-up cognition in this study indicates that even a short to moderate time interval is adequate for a substantial change in gait speed to impact on cognition. A longer follow-up period may have demonstrated an even greater association between changes in gait speed and changes in cognition in this cohort, as has been demonstrated previously [5].

The large sample size, covering a wide geographical area equivalent of 22% of the entire Nigerian population, allowed for a broad generalization of our findings. Those who dropped-out were older, and had the worst cognitive performance. We also purposively excluded all patients with a dementia diagnosis at baseline which may have led to an underestimation of the magnitude of the relationship between baseline gait speed and cognition. The use of culturally adapted measures which have been validated in the study setting has increased the reliability of the information obtained. Moreover, because we obtained a wide range of information on the health and well-being of the participants, we were able to adjust for several important confounders. However, the 2-year follow-up period, as well as our failure to employ a more comprehensive assessment of cognition may have limited our ability to demonstrate some important differences, or replicate findings reported in some studies conducted in the West. Finally, it is possible that persons covering the 3-m distance might have slower speed than those doing the 4-m distance because the former might have a shorter distance to get up to speed.

In concluding, the finding that baseline gait speed is associated with both follow-up condition and changes in cognition is potentially important for efforts at early identification and intervention in cognitive disorders, particularly in developing countries where organized care for people with cognitive disorders is still rudimentary.

## Figures and Tables

**Table 1:** Results of linear regression analyses examining the relationship between cognition at follow-up and gait speed at baseline study.

Baseline characteristic	Follow-up cognition	Multiple regression		Adjusted Wald test^b^		
	Mean (se)^a^	Co-efficient estimate	95% CI	*F*^c^	df	*P*
Gait speed						
1	9.64(0.20)					
2	10.23 (0.23)	0.64	0.14, 1.15	7.05	3, 30	0.001
3	11.22 (0.28)	1.07	0.49, 1.64			
4	11.49 (0.32)	1.24	0.48, 2.00			

Age group						
60–64	9.19 (0.28					
65–69	9.58 (0.32)	0.28	−0.59, 1.16	5.41	4, 29	0.002
70–74	10.06 (0.20)	0.73	0.10, 1.37			
75–84	10.74 (0.25)	1.25	0.53, 1.96			
85+	11.38 (0.24)	1.28	0.51, 2.04			

Sex						
Male	10.07 (0.23)					
Female	10.45 (0.17)	−0.08	−0.45, 0.30	0.17	1, 32	0.68

Location						
Urban	10.35 (0.30)					
Semi-urban	10.21 (0.24)	0.01	−0.62, 0.64	0.02	2, 31	0.98
Rural	10.16 (0.20)	0.05	−0.46, 0.56			

Current marital status						
Married	9.95 (0.18)					
Un-married	10.94 (0.20)	−0.46	−0.89, −0.04	4.96	1, 32	0.03

Economic status						
Low	10.65 (0.30)					
Low average	10.31 (0.17)	−0.06	−0.64, 0.52	0.63	3, 30	0.60
High average	10.14 (0.23)	0.20	−0.41, 0.81			
High	9.85 (0.27)	0.02	−0.66, 0.69			

Health status						
Very good	9.92 (0.25)					
Good	10.38 (0.18)	0.23	−0.20, 0.67	0.64	2, 31	0.53
Fair/poor	10.85 (0.34)	0.19	−0.46, 0.84			

Depression within the past 12 months						
Yes	10.77 (0.55)	0.27	−0.85, 1.39	0.24	1, 32	0.63
No	10.20 (0.18)					

Baseline word recall	0.38^d^	0.36	0.29, 0.43	98.07	1, 32	<0.0001
Change in gait speed 2007–2008^e^	−0.03^d^	0.09	−0.20, 0.38	0.38	1, 29	0.54
Change in gait speed 2007–2009^e^	0.005^d^	0.30	0.09, 0.51	8.59	1, 30	0.006

Gait speed categories: (1) <4.82 s 4 m; <3.62 s 3 m; (2) 4.82–6.20 s 4 m; 3.62–4.65 s 3 m; (3) 6.21–8.70 s 4 m; 4.66–6.52 s 3 m (4) >8.70 s 4 m; >6.52 s 3 m; unable to walk.

a Adjusted for sampling design but no covariates.

b Overall test of variable adjusted for sampling design.

c *F* test.

d Correlation coefficient.

e Results from supplementary models including change in gait speed; details not shown.

**Table 2:** Longitudinal analyses (2007, 2008, and 2009): random effects model showing the relationship between gait speed and cognition.

Variable	Co-efficient estimate	95% CI	Adjusted Wald test^a^		
			*χ*^2^	df	*P*
Year					
2007^b^					
2008	0.30	−0.06, 0.65			
2009	0.38	0.11, 0.65	8.67	2	0.01

Gait speed					
1^b^					
2	0.01	−0.26, 0.29			
3	0.28	−0.14, 0.70			
4	0.77	0.43, 1.2	27.7	3	<0.0001

Age group					
60–64^b^					
65–69	0.28	−0.04, 0.60			
70–74	0.39	−0.02, 0.81			
75–84	0.84	0.49, 1.2			
85+	1.3	0.95, 1.7	99.2	4	<0.0001

Sex					
Male^b^					
Female	−0.15	−0.42, 0.11	1.3	1	0.26

Location					
Urban^b^					
Semi-urban	0.15	−0.21, 0.51			
Rural	−0.17	−0.54, 0.20	2.96	2	0.23

Current marital status					
Married^b^					
Un-married	−0.55	−0.80, −0.30	18.9	1	<0.0001

Economic status					
Low^b^					
Low average	−0.32	−0.67, 0.03			
High average	−0.31	−0.75, 0.13			
High	−0.83	−1.4, −0.27	8.78	3	0.03

Health status					
Very good^b^					
Good	0.40	0.15, 0.65			
Fair/poor	0.69	0.27, 1.1	12.61	2	0.002

Depression within the past 12 months					
Yes^b^					
No	−0.02	−0.52, 0.48	0.01	1	0.94

BMI					
<18.5^b^	−0.21	−0.48, 0.06			
18.5–24.9	−0.31	−0.72, 0.10			
25–29.9	−0.49	−0.91, 0.0002	4.09	3	0.25
≥30					

Ever smoked^c^	−0.06	−0.27, 0.15	0.31	1	0.58
Ever drank alcohol^c^	−0.44	−0.70, −0.18	11.3	1	0.0008
Diabetes^c^	−0.75	−1.4, −0.10	5.18	1	0.023
Arthritis^c^	0.33	−0.02, 0.64	4.47	1	0.035
High blood pressure^c^	0.20	−0.03, 0.44	2.89	1	0.089
Stroke^c^	0.72	0.12, 1.3	5.46	1	0.020
COPD^c^	0.11	−0.33, 0.55	0.25	1	0.62

Gait speed categories: (1) <4.82 s 4 m; <3.62 s 3 m; (2) 4.82–6.20 s 4 m; 3.62–4.65 s 3 m; (3) 6.21–8.70 s 4 m; 4.66–6.52 s 3 m; (4) >8.70 s 4 m; >6.52 s 3 m; unable to walk.

a Overall test of variable using robust standard errors.

b Reference category.

c Relative to those without condition.

## References

[1] Liu-Ambrose T., Ahamed Y., Graf P., Feldman F., Robinovitch S.N. (2008). Older fallers with poor working memory overestimate their postural limits. Arch Phys Med Rehabil.

[2] Brach J.S., Hornyak V., VanSwearingen J.M. (2012). Measurement of gait speed. Top Geriatr Rehabil.

[3] Stuss D.T., Alexander M.P. (2000). Executive functions and the frontal lobes: a conceptual view. Psychol Res.

[4] Schneider J.A., Li J.L., Li Y., Wilson R.S., Kordower J.H., Bennett D.A. (2006). Substantia nigra tangles are related to gait impairment in older persons. Ann Neurol.

[5] Buracchio T., Dodge H.H., Howieson D., Wasserman D., Kaye J. (2010). The trajectory of gait speed preceding mild cognitive impairment. Arch Neurol.

[6] Gureje O., Kola L., Afolabi E. (2007). Epidemiology of major depressive disorder in elderly Nigerians in the Ibadan study of ageing: a community-based survey. Lancet.

[7] Welsh K.A., Butters N., Mohs R.C., Beekly D., Edland S., Fillenbaum G. (1994). The consortium to establish a registry for Alzheimer's disease (CERAD). Part V. A normative study of the neuropsychological battery. Neurology.

[8] Kessler R.C., Ustun T.B. (2004). The World Mental Health (WMH) survey initiative version of the World Health Organization (WHO) composite international diagnostic interview (CIDI). Int J Methods Psychiatr Res.

[9] American Psychiatric Association (1994). Diagnostic and statistical manual of mental disorders.

[10] National Health Statistics (1994).

[11] W. H. Organisation (1995). Physical status: use and interpretation of anthropometry.

[12] Verghese J., Robbins M., Holtzer R., Zimmerman M., Wang C., Xue X. (2008). Gait dysfunction in mild cognitive impairment syndromes. J Am Geriatr Soc.

[13] Gureje O., Ogunniyi A., Kola L., Abiona T. (2011). Incidence of and risk factors for dementia in the Ibadan study of aging. J Am Geriatr Soc.

